# Mating patterns and post-mating isolation in three cryptic species of the *Engystomops petersi* species complex

**DOI:** 10.1371/journal.pone.0174743

**Published:** 2017-04-07

**Authors:** Paula A. Trillo, Andrea E. Narvaez, Santiago R. Ron, Kim L. Hoke

**Affiliations:** 1 Museo de Historia Natural, Universidad Nacional Mayor de San Marcos, Lima, Perú; 2 Smithsonian Tropical Research Institute, Balboa, Ancon, Panama; 3 Department of Biology, Gettysburg College, Pennsylvania, United States of America; 4 Museo de Zoología, Escuela de Biología, Pontificia Universidad Catolica del Ecuador, Quito, Ecuador; 5 Department of Ecology, Environment and Evolution, La Trobe University, Bundoora, Australia; 6 Department of Biology, Colorado State University, Fort Collins, Colorado, United States of America; University of Arkansas, UNITED STATES

## Abstract

Determining the extent of reproductive isolation in cryptic species with dynamic geographic ranges can yield important insights into the processes that generate and maintain genetic divergence in the absence of severe geographic barriers. We studied mating patterns, propensity to hybridize in nature and subsequent fertilization rates, as well as survival and development of hybrid F_1_ offspring for three nominal species of the *Engystomops petersi* species complex in Yasuní National Park, Ecuador. We found at least two species in four out of six locations sampled, and 14.3% of the wild pairs genotyped were mixed-species (heterospecific) crosses. We also found reduced fertilization rates in hybrid crosses between *E*. *petersi* females and *E*. *“magnus”* males, and between *E*. *“magnus”* females and *E*. *“selva”* males but not in the reciprocal crosses, suggesting asymmetric reproductive isolation for these species. Larval development times decreased in F_1_ hybrid crosses compared to same species (conspecific) crosses, but we did not find significant reduction in larval survival or early metamorph survival. Our results show evidence of post-mating isolation for at least two hybrid crosses of the cryptic species we studied. The general decrease in fertilization rates in heterospecific crosses suggests that sexual selection and reinforcement might have not only contributed to the pattern of call variation and behavioral isolation we see between species today, but they may also contribute to further signal divergence and behavioral evolution, especially in locations where hybridization is common and fertilization success is diminished.

## Introduction

The rate of discovery of cryptic species has increased exponentially over the past two decades due to advances that allow more thorough genetic sampling [1–3]. These studies continue to uncover complexes of closely related species with minimal phenotypic differences, but with small, patchy distributions, overlapping ranges, and distinctly different mitochondrial lineages [4–8].

Determining the extent of reproductive isolation in cryptic species not separated by large geographical barriers is especially important, because in such cases, geographic ranges are often highly dynamic, allowing species to separate from and come back into contact with one another. Understanding the propensity to hybridize in nature, subsequent fertilization rates, and development of hybrid offspring can give us windows into the cohesiveness or fluidity of these species, as well as insights into the processes that lead to the origins and maintenance of genetic divergence in the absence of conspicuous morphological or habitat differentiation [9,10].

Although genetic surveys can provide a snapshot in time, revealing the extent of cryptic species in a specific clade, it is important to accompany these surveys with studies of current overlapping ranges and hybridization propensity, as well as measurements of pre and post-mating reproductive isolation. In this study, we determined range overlap and the level of post-mating reproductive isolation for three cryptic species within the *Engystomops petersi* species complex. While one of these species corresponds to the previously described *E*. *petersi* (Jiménez de la Espada, 1872), the other two species have been classified as confirmed candidate species but remain undescribed [4,11,12]. We will refer to these last two species as *E*. “selva” (= Clade D in Funk et al. 2011) and *E*. “magnus”, (= Clade A in Funk et al. 2011).

Frogs of the *Engystomops petersi* species complex (also known as *Physalaemus petersi*) are nocturnal, pond-breeding, and found in rainforest habitats throughout the Amazon Basin [13–15]. In a systematics study, Funk *et al. [4]* found that the two recognized species in this complex (*E*. *petersi* and *E*. *freibergi*) are actually at least seven cryptic species. Within this species complex, *E*. *“selva”* and *E*. *“magnus”* are more closely related to each other than to *E*. *petersi* [4]. This genetic divergence predicts divergence in mating calls across the *Engystomops petersi* complex [16] and calls of the three species differ in syllable number and spectral properties [17,18].

Behavioral isolation based on female choice of call properties occurs between some but not all of the three species studied here. Females from *E*. *“magnus”* significantly prefer calls from males of their own species to *E*. *“selva”* male calls in two-choice preference experiments, and females of *E*. *“selva”* also prefer conspecific calls to those of *E*. *“magnus”* [19]. Females of *E*. *petersi* strongly discriminate against *E*. *“selva”* calls in favor of calls from male conspecifics, but they display phonotaxis to calls of males of *E*. *“magnus”*, which have similar spectral properties [17]. Together, these results suggest that female choice and behavioral isolation in these species are consistent with spectral differences in male courtship signals. Based on these acoustic preference tests, it has been hypothesized that sexual selection or reinforcement have driven mating call divergence and speciation in the *E*. *petersi* species complex [17,19]. Additionally, geographic variation in predation pressure likely also plays a role in the maintenance of signal divergence within this complex [20]. The extent to which these species overlap and mate, however, as well as the importance of post-mating reproductive isolation between them are not well known.

Here, we investigate the role of post-mating reproductive isolation on the maintenance of species boundaries in the *E*. *petersi* species complex. We describe the existence of small scale species range overlap and the prevalence of heterospecific mating in nature. We also measured fertilization success as well as tadpole development and survival of F_1_ hybrid crosses for *E*. *petersi*, *E*. *“magnus”*, and *E*. *“selva”*. Finally, we discuss our results in light of previous studies of behavioral isolation in these species and address the importance of post-mating reproductive isolation in the *E*. *petersi* species complex.

## Methods

### Field collections

Reproductive adults were collected at night in temporary ponds. Six different sites were sampled in Yasuní National Park, Orellana Province, between September, 2009 and July, 2011 (see S1 Table for site coordinates)

To assess hybridization in nature, we collected all mating pairs (hereafter called pairs in amplexus or amplectant pairs). We also collected a subset of calling males as well as any single females encountered to further characterize species composition at each site. Frogs were used in experimental crosses or maintained in a captive colony for use in other experiments. Individuals were identified using photographs of their ventral surfaces. We determined species composition of each site and each amplectant pair using genetic barcoding for each frog as described below.

### Experimental crosses

We compared fertilization success between conspecific and heterospecific matings by conducting breeding experiments in captivity. All experimental crosses and rearing took place in a custom-built enclosure at the Yasuni Research Station using rearing methods adapted from those described for *E*. *randi* [21]. The enclosure maintained terraria at ambient humidity levels and understory temperatures. Terraria were illuminated by indirect sunlight and sprayed with rainwater as necessary to maintain substrate moisture.

Gravid females from the Yasuní area were crossed the same night they were captured in amplexus to ensure reproductive condition. We selected males for breeding that had been calling in the captive colony for several days prior to the cross attempt. Species identification occurred months after crossing, thus all breeding experiments using Yasuní area frog pairs were completed with experimenters blind to cross type. On the other hand, *E*. *“selva”* frogs from La Selva Lodge were transported to Yasuní and mated with Yasuní frogs, so investigators knew the species identification of some *E*. *“selva”* individuals. Individuals to be crossed were introduced into plastic terraria (25 x 25 x 40 cm) containing a thin layer of rocks and leaf litter, and with a small pool of rainwater formed by creating a depression in the rocks (at least 1 cm depth). The plastic lid for the terrarium was modified for better ventilation by replacing the plastic with mesh. Females and either one or two males were housed together until they mated, or for 10 days if no mating occurred. In terraria with two males, if one male was found in amplexus, the other was removed, and mating was allowed to continue. While in terraria, frogs were fed termites *ad libitum*.

**Fig 1:**
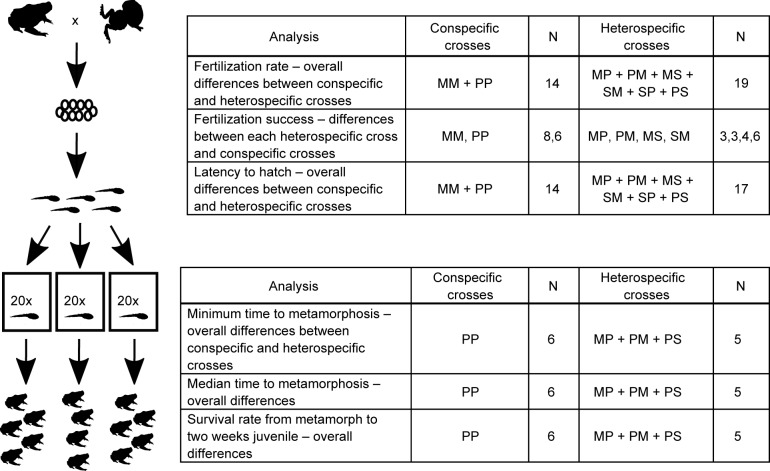
Rearing methodology and description of analyses. (A) Within 12 hours after mating, we transferred foam nests to a container with 5cm deep rainwater. On the 5^th^ day after the first tadpole hatched, we separated tadpoles into three rearing tanks containing 20 tadpoles each. (B) Table including the list of crosses used for each analysis with sample size for each group. Single numbers represent the total number of crosses for that group combination; numbers separated by a comma represent sample sizes for each cross type in the same order in which the cross types are presented.

For successful crosses, within 12 hours after mating, we transferred the foam nest to a plastic tub (25 x 25 x 40 cm) containing 5 cm deep rainwater. Nests were checked at least twice a day. As soon as the first tadpole emerged, we oxygenated the water with aquarium pumps and fed tadpoles *ad libitum* with Super Alimento de Renacuajos (SAR: protein– 27%, humidity = 10.02%, fiber = 42.66% and Carbohydrates = 48.82% [21,22]). Latency to hatch was recorded as the number of days it took for the first tadpoles to emerge from the nest. For each cross, we removed the foam nest from the water five days after the first tadpole emerged, separated and counted unfertilized eggs and subsequently separated the tadpoles into three rearing tanks containing 20 tadpoles each (Fig 1). Tadpoles were fed SAR daily, and water was changed at least once a week with fresh rainwater. We counted tadpoles daily and recorded developmental stage. For each tadpole, we recorded the date all four legs emerged (time of metamorphosis), and transferred the metamorph to a transition terrarium (20 cm diameter x10 cm high) with a pool of water on one side and gravel and plants on the other. We maintained metamorphs while feeding them SAR and termites for an additional 14 days after leaving the water to chart juvenile survival. We did not include conspecific *E*. *“selva”* crosses in the analyses because we only had one cross (see Fig 1 for a list of crosses used in each analysis).

### Species identification

To determine the “species” of each individual, we used one fragment of mitochondrial DNA that uniquely identifies each species [4]. We sequenced a fragment of ~850 bp of the genes tRNA-Val and 16S RNA (partial sequence) using primers GGCAAGTCGTAACATGGTAAG (forward, [23]) and ATGTTTTTGGTAAACAGGCG (reverse [24]). We selected outgroup species (*E*. *pustulatus*, *E*. *coloradorum*, and *E*. *pustulosus*) based on previous phylogenetic analysis [25]. Following Geneious Pro 5.4.6 alignment [26], we imported the matrix into GARLI version 2.0 [27] to construct phylogenies using maximum-likelihood (ML). The analysis included all sequences from Funk et al. 2011 [4]. All sequences fell into well-supported clades (bootstrap values > 90) corresponding to each of the three species, and species assignment was unambiguous. All the species identification was done at the Museo de Zoología, Pontificia Universidad Católica del Ecuador (QCAZ).

### Animal collection and ethics statement

To avoid introduction and transfer of novel genetic background into wild populations and to sample genetic material, all individuals used in this study were euthanized and tissue for DNA extraction was collected from the liver. Methods conformed to the AVMA 2013 Guidelines for the Euthanasia of Animals. All voucher specimens were deposited at QCAZ. Animal collection and research permits were provided by the Ecuadorian Ministerio de Ambiente (No.008-09 IC-DNB/MA, N o.001-10 IC-FAU-DNB/MA and No.0032-DPO-MA). This research was approved by the Institutional Animal Care and Use Committee at Colorado State University (09-027A-01).

### Statistics

All statistical analyses were conducted in SAS 9.0 (SAS Institute Inc., Cary, NC, USA). We used a linear mixed model to compare the proportion of eggs that were fertilized among cross types. The independent variable in the overall model was a factor indicating each unique cross type (species ID of dam and species ID of sire). We estimated specific planned comparisons as posthoc analyses to determine the cross types with low fertilization success. First, we analyzed overall differences between conspecific and heterospecific crosses in fertilization success by comparing all of six heterospecific cross types combined vs. the two conspecific crosses combined (*E*. *“magnus”-E*. *“magnus”* and *E*. *petersi-E*. *petersi*). A second contrast was performed between each heterospecific cross type and the two conspecific crosses. For the latter, we did not conduct the posthoc analyses of the two heterospecific cross directions between *E*. *“selva”* and *E*. *petersi* due to limited sample sizes. We used generalized linear models with a negative binomial distribution and a log-link function to estimate the influence of cross type on latency to hatch, the minimum and the median days between hatching and metamorphosis (number of days to the first and to the median metamorph), and survival rate to two-week old metamorphs. Analyses of latency to hatch included conspecific *E*. *“magnus”* and *E*. *petersi* crosses as well as all six heterospecific crosses. However, due to differences in husbandry, we only used *E*. *petersi* conspecific crosses and heterospecific *E*. *petersi* and *E*. *“magnus”* or *E*. *petersi* and *E*. *“selva”* crosses for analyses of time to metamorphosis and survival rate (Fig 1).

## Results

### Overlapping range and hybridization in nature

**Fig 2:**
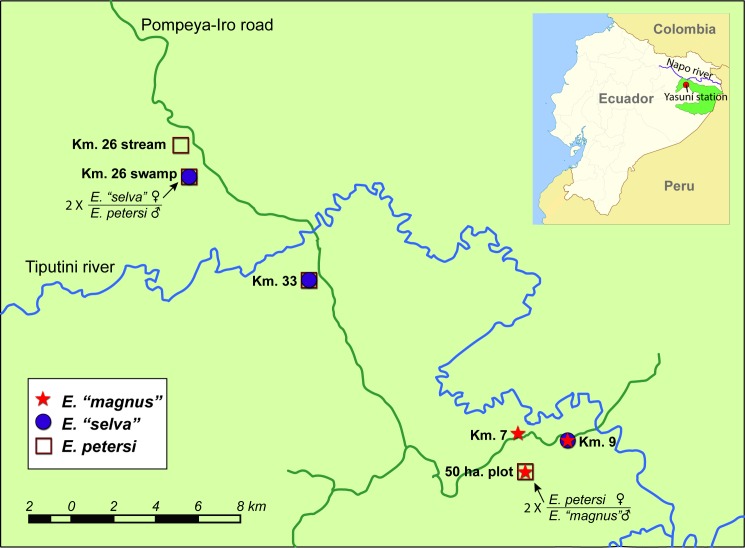
Map of sampling sites at Yasuní National Park. Star symbols indicate presence of *E*. *“magnus”*, circles indicate presence of *E*. *“selva”*, and squares indicate presence of *E*. *petersi*. Sites with hybrid pairs are shown with an arrow.

Of the six sites sampled in Yasuni National Park, we found four sites with at least two reproductive species in them (Fig 2). Two sites had both *E*. *petersi* and *E*. *“selva”*, one had *E*. *“selva”* and *E*. *“magnus”*, and a fourth site had *E*. *petersi* and *E*. *“magnus”* individuals. At some sites, two species were found calling in the same chorus, further increasing the opportunities for hybridization. Of the 28 amplectant pairs collected with complete genetic identification, a total of four pairs were heterospecific pairings, two consisting of female *E*. *“selva”* paired with male *E*. *petersi* in one site (Km 26-swamp site, Fig 2), and two with female *E*. *petersi* and male *E*. *“magnus”* in another site (50ha plot site, Fig 2). In each case, the female was the rarest species at that local chorus (10% or 2 out of 20 genotyped females were *E*. *“selva”* in the Km26-swamp site and 31% or 4 out of 13 genotyped females were *E*. *petersi* in the 50ha plot site).

### Fertilization success

**Fig 3:**
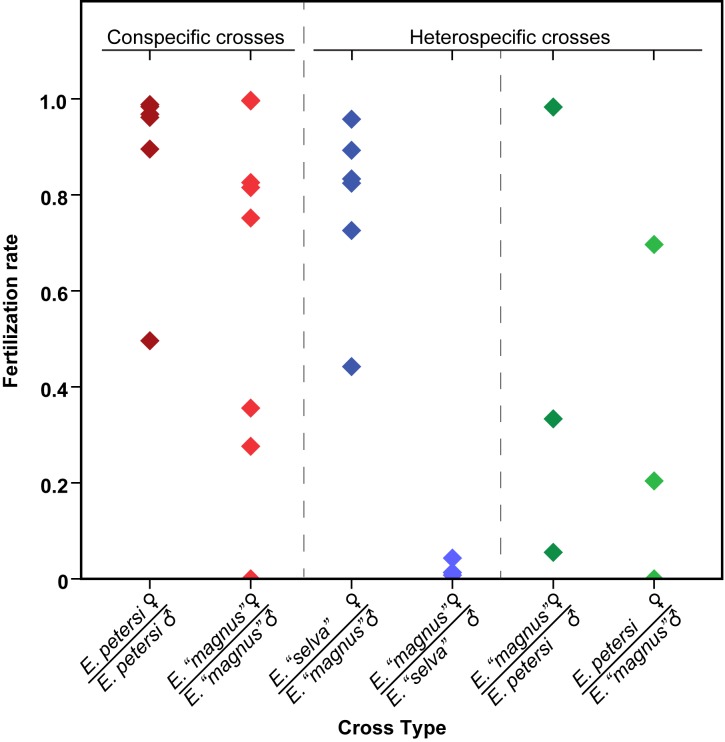
Fertilization rates for conspecific and heterospecific crosses. Overall fertilization rates decreased in heterospecific crosses compared to conspecific crosses but this was due to significant reductions in only some of the heterospecific cross directions.

There was a significant effect of cross type on fertilization success in crosses within the *E*. *petersi* species complex (ANOVA, F_7,27_ = 4.13, *P* = 0.003). We found reduced fertility in heterospecific crosses compared to conspecific crosses (ANOVA planned contrasts, F_1,27_ = 6.01, *P* = 0.021). However, this reduced fertility in heterospecific crosses was driven by significant reductions in only some of the crosses. Both *E*. *petersi* female-*E*. *“magnus”* male crosses (PM) and *E*. *“magnus”* female-*E*. *“selva”* male crosses (MS) showed reduced fertilization rates when compared to conspecific crosses (least square mean fertilization rate ± standard error for PM = 0.3 ± 0.17 and MS = 0.02 ± 0.15; planned contrasts for PM vs. conspecific: F_1,27_ = 5.77, *P* = 0.023, and MS vs. conspecific: F_1,27_ = 18.95, *P* = 0.0002, Fig 3). However, *E*. *“magnus”* female-*E*. *petersi* male (MP) and *E*. *“selva”* female-*E*. *“magnus”* male (SM) crosses did not show any reduction in fertilization rate (least square mean fertilization rate ± standard error for MP = 0.46 ± 0.17, and SM = 0.81 ± 0.11; planned contrasts for MP vs. conspecific: F_1,27_ = 2.49, *P* = 0.126, and SM vs. conspecific: F_1,27_ = 0.15, *P* = 0.705; Fig 3).

### Development and survival

Heterospecific and conspecific crosses did not differ in the latency to hatch (generalized linear model with negative binomial distribution and log-link function, χ^2^ = 0.2846, df = 2, *P* = 0.285). However, minimum time to metamorphosis was reduced in heterospecific crosses when compared to conspecific *E*. *petersi* crosses (mean time ± SD for heterospecific crosses = 51.34 ± 16.86 days; mean time ± SD for conspecific crosses = 76.89 ± 12.308 days; χ^2^ = 4.39, df = 1, *P* = 0.036). Median time to metamorphosis was also significantly lower in heterospecific crosses when compared to conspecific crosses (average median time ± SD for heterospecific crosses = 75.16 ± 31.08 days; average median time ± SD for conspecific crosses = 114.71 ± 25.29 days; χ^2^ = 4.1, df = 1, *P* = 0.043). Finally, we found no difference in survival rate to two-week old metamorphs between heterospecific and conspecific crosses.

## Discussion

Our study shows that *E*. *petersi*, *E*. *“selva”*, and *E*. *“magnus”* overlap extensively in Yasuni National Park, and this range overlap has led to heterospecific pairings in nature. Whether heterospecific pairings lead, in turn, to successful hybridization will depend, in part, on the fertilization success and development of offspring in these crosses. Our results show that both fertilization success and tadpole developmental time differ significantly in heterospecific crosses compared to conspecific crosses in the *E*. *petersi* species complex.

We found reduced fertilization rates in both of the heterospecific crosses we conducted (*E*. *“magnus”*- *E*. *petersi* and *E*. *“magnus”*- *E*. *“selva”*). However, some hybrid cross directions had low success, while others were relatively fertile. Asymmetric post-mating isolation is a common occurrence in hybridizing species of amphibians [28], and can result from incompatibilities between heterogametic sex chromosomes, incompatibilities in nuclear-mitochondrial genomes, or from maternal epigenetic effects [29]. While our study does not address the possible mechanisms of post-mating isolation in the *E*. *petersi* complex, karyotypic differences between the species present themselves as a promising area for future research. Although all three species have the same chromosome number (2n = 22), cytogenetic analyses have revealed high variation in chromosomal structure (including centromere position, banding patterns and length), as well as presence or absence of heteromorphic sex chromosomes [30]. Heteromorphic sex chromosomes are a rare condition in anurans, yet they are present in *E*. *petersi* and not in *E*. *“selva”* or *E*. *“magnus”* [30,31]. Hybrid offspring of species with very different karyotypes may have unbalanced genomes and reduced fitness. Thus, karyotypic differences between *E*. *petersi*, *E*. *“magnus”* and *E*. *“selva”* could be an important factor influencing fertilization success and the development of reproductive isolation [30].

Our study also revealed a significant decrease in tadpole development time (both minimum and median time to metamorphosis) in heterospecific crosses when compared to conspecific crosses, regardless of cross direction. Faster developmental times in frogs have been suggested alternatively as a sign of heterozygote advantage, adaptive responses to environmental cues, or as the result of high physiological stress [32–35]. Ecologically speaking, reduced developmental times can be beneficial if they lower risks posed by predation or desiccation [36], but they can also lead to ecological and physiological tradeoffs in the later developmental stages [37–39]. Studies examining the full lifespan of hybrid F_1_ offspring in the *E*. *petersi* species complex will be necessary to resolve the importance, if any, of this reduction in developmental timing for offspring’s fitness.

We found no differences between heterospecific and conspecific crosses neither in latency to hatch nor in tadpole survival for two weeks after metamorphosis. Tadpole survival was, however, highly variable across crosses, and this variability may have obscured any small differences in survival due to cross type. F_1_ individuals with high survival could still have reduced fertility if karyotypic differences generate unbalanced genomes that are lethal for F_2_ zygotes. In a separate experiment, we attempted to breed F_2_ MS hybrid and MM pure lines and we observed a wide range of fertilization rates for the pure line, 0–90%, as compared to very low fertilization (0–4%) for the hybrid line. These low fertilization rates in the hybrid line eventually produced a total of four F_2_ hybrid individuals (unpub. data).

Although our study shows selection against heterospecific crosses in the *E*. *petersi* complex, we also found that amplectant frogs at our field sites included heterospecific pairs. This, along with our result that some crosses produce high hatching rates, suggests a potential for the presence of F_1_ hybrid individuals in nature. Although Funk et al. (2011) suggested that hybridization among these species involves ancient mitochondrial introgression from *E*. *“magnus”* into *E*. *“selva”*, studies determining the prevalence and abundance of F_1_ hybrids in nature [40] as well as whether they are fertile or not, will add to our understanding of the nature and extent of the interactions between these cryptic species. Ultimately, low fertilization rates of hybrid crosses and karyotypic differences that prevent fertility of F_1_ hybrids may be sufficient to maintain reproductive isolation in sympatric populations.

**Fig 4:**
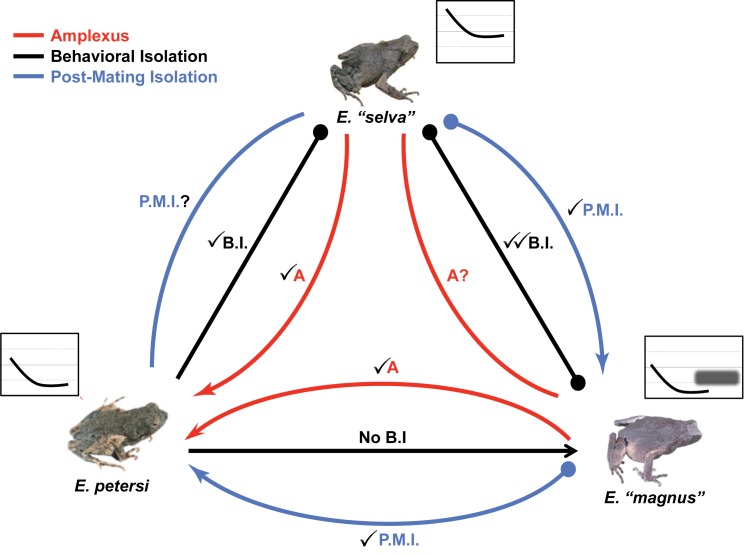
Schematic of species interactions for *E*. *petersi*, *E*. *“magnus”* and, *E*. *“selva”*. Summarizes observations of pairs observed in hybrid amplexus and post-mating isolation results from the current study, along side previously reported results from behavioral isolation trials (Guerra and Ron, 2008; Boule et al, 2007). Arrows indicate cross direction or high fertilization success. Filled circles indicate direction of rejection during behavioral trials or decrease in fertilization success (female → male).

In the *E*. *petersi* species complex, pre-mating isolation and post-mating fertilization success are not always associated. *E*. *“selva”* and *E*. *“magnus”* show behavioral isolation due to differences in the males’ calls when tested in laboratory assays [19] and we did not find them in cross-species amplexus. The low fertilization rate in the *E*. *“magnus”*-*E*. *“selva”* crosses matches this strong behavioral isolation, and hybrids, therefore, may be very rare despite overlapping populations in at least one breeding site (Fig 4). In contrast, while *E*. *petersi* and *E*. *“selva”* also differ in the spectral content of their calls and display behavioral isolation, we found two pairs in cross-species amplexus at sites where these species overlap (Fig 4). In each case, the *E*. *“selva”* female was in amplexus with a heterospecific male from the locally abundant *E*. *petersi* species. Unfortunately, our heterospecific crosses for these species were too few to include them in the posthoc analysis of fertilization rates, so we cannot predict the outcome of these matings. Finally, *E*. *petersi* and *E*. *“magnus”* produce calls with similar spectral content and do not show behavioral isolation [17,40]. Consistent with their lack of spectral differences and lack of behavioral isolation, we found two pairs in cross-species amplexus. Importantly, our study shows that heterospecific crosses between *E*. *petersi* and *E*. *“magnus”* have reduced fertilization success and shorter tadpole developmental time (Fig 4). Thus, our results suggest that a lack of behavioral isolation between these species can result in detrimental hybridization.

This study shows evidence of small-scale range overlap, heterospecific matings, and post-mating isolation within the *E*. *petersi* species complex. Interestingly, we find a pattern of post-mating isolation for crosses between *E*. *petersi* and *E*. *“magnus”* species, which did not display behavioral isolation in previous studies. Overlap in distribution of *E*. *petersi* and *E*. *“magnus”* may be recent, or genetic variability for mating behaviors may not be sufficient, for selective pressures against hybrids to drive divergence in female preferences and male signals in this species pair. While previous studies have explored mechanisms of behavioral isolation between these frogs, the documentation of hybrid pairs in nature, and the quantification of fitness costs when hybrid matings do occur gives us a window into the cohesiveness or fluidity of this species complex. As cryptic species with dynamic geographic ranges continue to be discovered, studies that look at post-mating isolation as well as hybrid mating in nature can give us a fuller picture of the dynamics of the reproductive frontiers that define these species.

## Supporting information

S1 Table Names and coordinates of sites sampled in Yasuní National Park, Orellana, Ecuador. (DOCX)
