# Supplementary material for: Mating patterns and post-mating isolation in three cryptic species of the Engystomops petersi species complex
Source: PLoS One. 2017 Apr 7;12(4):e0174743. doi: 10.1371/journal.pone.0174743 (PMC5384746; doi:10.1371/journal.pone.0174743)
Supplement: S1 Table — (DOCX) [file pone.0174743.s001.docx]

**S1 Table.** **Names and coordinates of sites sampled in Yasuní National Park, Orellana, Ecuador**

| **Site Name** | **South** | **West** | **Elevation (m)** |
| --- | --- | --- | --- |
| **Km. 7_ECY Road** | 0° 40.703’ | 76° 23.935’ | 244 |
| **Km. 9_ECY Road** | 0° 40.723’ | 76° 23.091’ | 233 |
| **50 ha. Plot _ECY** | 0° 41.110’ | 76° 23.900’ | 221 |
| **Km. 26 Stream_Pompeya-Iro Road** | 0° 34.755' | 76° 30.674’ | 255 |
| **Km. 26 Swamp_Pompeya-Iro Road** | 0° 35.093’ | 76° 30.369’ | 217 |
| **Km. 33_Pompeya-Iro Road** | 0°37.386’ | 76°27.993’ | 227 |

***ECY = Estación Científica Yasuní**
